# Structure, Toxicity, Prevalence, and Degradation of Six Understudied Freshwater Cyanopeptides

**DOI:** 10.3390/toxins17050233

**Published:** 2025-05-08

**Authors:** Blake B. Stringer, Regina G. Szlag Silva, Jeremy J. Kodanko, Judy A. Westrick

**Affiliations:** Department of Chemistry, Wayne State University, Detroit, MI 48202, USA; bstrings@wayne.edu (B.B.S.); regina.szlag@wayne.edu (R.G.S.S.); jkodanko@wayne.edu (J.J.K.)

**Keywords:** cyanopeptides, anabaenopeptins, cyanopeptolins, aeruginosamides, aeruginosins, microginins, cyclamides, microcystins, cyanobacterial harmful algae blooms

## Abstract

Anthropogenic influences have increased global warming and eutrophication, escalating the frequency and severity of harmful cyanobacterial blooms (cHABs) in freshwater ecosystems. These blooms release cyanopeptides, a diverse class of bioactive compounds with varying acute and chronic toxicities upon ingestion. To date, research has prioritized acutely toxic cyanopeptides like microcystins. As a result, significantly less is known about other freshwater cyanopeptides. This review highlights six understudied cyanopeptide classes, anabaenopeptins, cyanopeptolins, aeruginosamides, aeruginosins, microginins, and cyclamides, and provides a comprehensive overview of their molecular structures, toxicological profiles, environmental concentrations, and known degradation pathways. Given the potential toxicity, increased environmental abundance, and environmental stability of many cyanopeptides in freshwater sources, further research is needed to understand if degraded cyanopeptides are still biologically active prior to entering drinking water to ensure public health.

## 1. Introduction

The increasing frequency of cyanobacterial harmful algae blooms (cHABs) in freshwater sources poses a significant threat to drinking water safety worldwide [1]. While cyanobacteria play essential roles in biogeochemical cycling, metal transformation, and food web support, their uncontrolled proliferation leads to severe ecological and public health consequences [2]. cHABs contribute to hypoxic zones, biodiversity loss, and the production of cyanopeptides, which can cause adverse health effects ranging from mild gastrointestinal distress to life-threatening liver failure [3,4,5]. The rising incidence of cHABs is primarily driven by anthropogenic influence, resulting in global warming and nutrient enrichment from agricultural and urban runoff [6,7,8]. When cyanobacterial cells lyse, they release intracellular cyanopeptides into the water, necessitating monitoring, removal, and deactivation strategies at drinking water treatment plants (DWTPs) [9,10].

The recognition of acutely toxic cyanopeptides as significant drinking water contaminants first emerged in the 1990s, prompting regulatory agencies such as the US Environmental Protection Agency to include several cyanopeptides, known as cyanotoxins (cylindrospermopsin, anatoxin-a, microcystins, and saxitoxins), on successive contaminant candidate lists. Microcystins (MCs), potent hepatotoxins, have received the most research attention due to their association with numerous animal and occasional human fatalities [4,11,12,13]. However, a single cHAB can produce a complex mixture of cyanopeptides, with no predictable pattern governing the co-production of these secondary metabolites [14,15,16]. Furthermore, the potential for synergistic or antagonistic interactions among cyanopeptides remains largely unexplored, though early studies suggest that co-exposure to MCs and other secondary metabolites may enhance toxicity [17,18,19,20,21].

**Figure 1:**
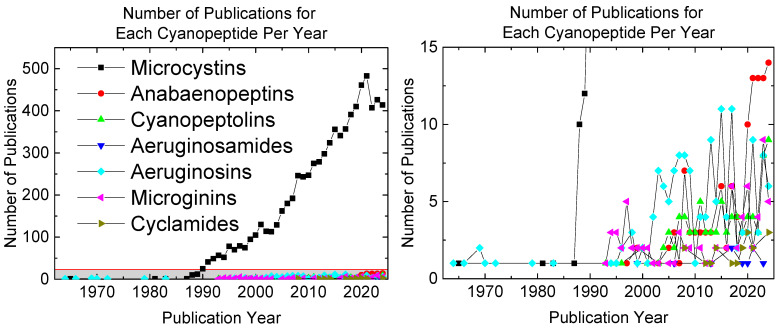
Comparison of the number of publications each year that mention the following cyanopeptides: microcystins, anabaenopeptins, cyanopeptolins, aeruginosamides, aeruginosins, microginins, and cyclamides.

Despite cyanopeptides’ possible toxicity and abundance, several remain understudied when compared to MCs (Figure 1). Historically, research efforts have focused on acutely toxic cyanopeptides like MCs, whereas chronic toxicity, environmental persistence, and degradation of other cyanobacteria secondary metabolites have been largely overlooked. The lack of resources placed toward other secondary metabolites has led to the lack of analytical standards, making toxicology studies and field analyses difficult. This review focuses on six understudied cyanopeptide classes (anabaenopeptins, cyanopeptolins, aeruginosamides, aeruginosins, microginins, and cyclamides) and examines their molecular structures, known toxicological profiles, known environmental prevalence, and potential deactivation before and at DWTPs. Given the widespread detection of cHABs in freshwater sources, understanding the fate of all cyanopeptides is critical for assessing human health risks and informing future regulatory decisions.

## 2. Cyanopeptide Structure, Toxicity, and Prevalence

The acutely toxic cyanopeptides, MCs, are cyclic heptapeptides with a variability of L-amino acids [22]. The non-proteinogenic amino acids critical for toxicity towards protein phosphatases are (2*S*,3*S*,4*E*,6*E*,8*S*,9*S*)-3-amino-9-methoxy-2,6,8-trimethyl-10-phenyldeca-4,6-dienoic acid (Adda), and *N*-methyldehydroalanine (Mdha). Adda binds with hydrophobic interactions in the enzyme binding pocket while Mdha forms a covalent linkage to a cysteine residue, further securing the toxin [23,24,25]. Anabaenopeptins, cyanopeptolins, aeruginosamides, aeruginosins, microginins, and cyclamides exhibit structural diversity, ranging from cyclic to linear peptides and incorporate a variety of proteogenic and non-proteogenic amino acid residues [26,27,28,29,30,31]. Their known toxicological effects include inhibition of serine proteases, phosphatases, carboxypeptidases, and peptidases, as well as cytotoxic and antiparasitic activity [32,33,34,35,36,37]. Additionally, these cyanopeptides have been detected up to ppm level concentrations in extreme cases, raising concerns about their persistence and potential resilience in DWTPs [38,39]. Table 1 provides an overview of each class’ respective toxicities and observed concentrations.

### 2.1. Anabaenopeptin’s Structure, Toxicity, and Prevalence

**Figure 2:**
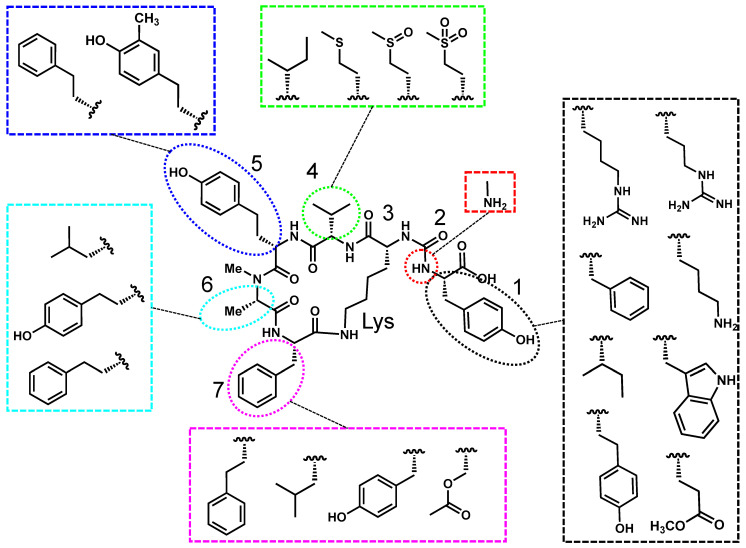
Anabaenopeptin A is used as a representative structure for all freshwater anabaenopeptins. The variable residues are shown in the colored boxes, and the positions are numbered 1–7.

Anabaenopeptins are typically named after the genera they were discovered in. Some examples of anabaenopeptins with differing nomenclature are lyngbyaureamide, nodulapeptin, and oscillamide [53,54,55]. Regardless, all anabaenopeptins constitute a diverse family of hexapeptides with a pentapeptide core, characterized by an invariant lysine residue and a unique ureido linkage at positions 2 and 3. As illustrated in Figure 2, the lysine and ureido groups are conserved across all congeners, while other structural positions exhibit significant variability. The ureido linkage, located at position 2, connects the α-amino group of a variable N-terminal amino acid (position 1) to the amino group of lysine, introducing conformational rigidity that is crucial for biological activity. The greatest variability occurs at position 1, where residues range from polar amino acids (e.g., arginine, tyrosine) to nonpolar amino acids (e.g., phenylalanine, isoleucine). Positions 4–7 further contribute to anabaenopeptin diversity, incorporating both proteinogenic and non-proteinogenic residues such as homotyrosine, homophenylalanine, acetyl serine, and methionine derivatives [34,40,53,56,57,58]. This variability has led to the identification of over 100 anabaenopeptin congeners, each with potentially unique toxicological properties [26].

Anabaenopeptins have traditionally been evaluated in comparison to MCs, whose hepatotoxicity primarily arises from the inhibition of protein phosphatases (PP1 and PP2A) [59]. These enzymes regulate essential cellular functions, including signal transduction, metabolism, and cell cycle progression. Anabaenopeptins A, D, F, and oscillimide Y inhibit PP1 with IC_50_ values that are nanomolar, while anabaenopeptin F, oscillimides A, B, and Y inhibit PP2A IC_50_ values in the micromolar range [33,41].

Beyond protein phosphatase inhibition, certain anabaenopeptins inhibit carboxypeptidase A (CPA) and carboxypeptidase B (CPB). Many congeners inhibit CPA in the low µM range, while anabaenopeptin G exhibits an IC_50_ of 1.9 nM [34]. Further increased potency is found with anabaenopeptin B, which inhibits CPB at 0.2 nM, with structural modeling confirming that the arginine at position 1 is critical for binding [43].

In addition to phosphatases and carboxypeptidases, anabaenopeptins also mildly inhibit serine proteases, though inhibition is highly congener-specific. For instance, anabaenopeptin MM850 inhibits trypsin (IC_50_ = 45 µM), while anabaenopeptins A and D do not inhibit trypsin. Similarly, chymotrypsin and elastase are inhibited by anabaenopeptins MM823, MM850, and oscillimide Y (IC_50_ < 50 µM), whereas other congeners (e.g., ferintoic acids A and B) lack inhibitory activity [40,55,57].

Anabaenopeptins rank among the most abundantly produced cyanopeptides in freshwater systems. They are synthesized by diverse cyanobacterial genera, including *Microcystis*, *Nodularia*, *Oscillatoria*, *Dolichospermum*, and *Planktothrix*, via the action of nonribosomal peptide synthetases (NRPS), *apn* [60,61,62,63]. For example, in Canadian lakes, MC levels have been reported above the World Health Organization’s drinking water guidelines, yet anabaenopeptin concentrations were found to be nearly three times higher (approximately 10 ppb) than those of MC [64]. In a Spanish reservoir, while MCs were detected only at the ppt level, intracellular and surface water concentrations of anabaenopeptin F reached record levels of 58 ppm and 1.1 ppm, respectively [38]. In North American waters, high intracellular levels of anabaenopeptin B (4.3 ppm) were observed, whereas MC levels remained below 1 ppb, which is below the drinking guidelines thresholds [36]. Notably, some cyanobacterial strains produce anabaenopeptins but not MCs. For instance, certain *Dolichospermum* and *Oscillatoria* strains synthesize anabaenopeptin D and NZ842 at low ppb levels in the absence of MC [42].

Anabaenopeptins exhibit considerable structural variability, with position 1 playing a key role in their mode of action [43]. Their strongest inhibitory activity is against carboxypeptidases, though they also exhibit moderate inhibition of protein phosphatases and serine proteases [33,34]. Despite their lower potency against certain enzyme targets, anabaenopeptins remain a potential health concern due to their high environmental concentrations, with levels reaching up to ppm concentrations in drinking water sources [38].

### 2.2. Cyanopeptolin’s Structure, Toxicity, and Prevalence

**Figure 3:**
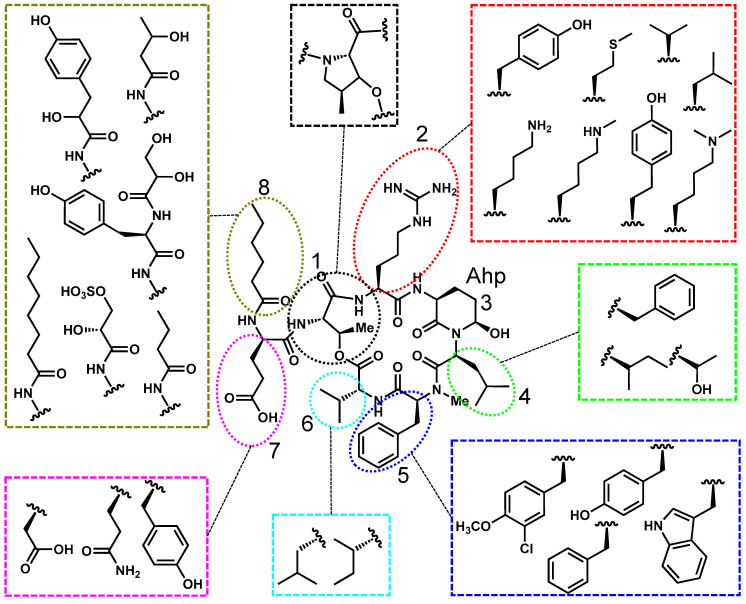
Cyanopeptolin A is used as a representative structure for all freshwater cyanopeptolins. The variable residues are shown in the colored boxes, and the positions are numbered 1–8.

Cyanopeptolins, also known as micropeptins and anabaenopeptilides, are a structurally diverse group of cyclic pentapeptides distinguished by their macrocyclic peptide backbone containing an ester linkage with variable exocyclic N- and C-terminal residues (Figure 3). A defining structural feature of cyanopeptolins is the 3-amino-6-hydroxy-2-piperidone (Ahp) moiety at position 3, which influences molecular conformation. Position 1 is typically threonine, though recent variants have replaced threonine with a cyclic amino acid, increasing structural diversity [65]. Positions 2, 5, and 8 are highly variable, with position 2 frequently occupied by tyrosine or arginine variants, which may significantly influence toxicological activity [66,67].

Cyanopeptolins are best known for their potent serine protease inhibition, disrupting protein degradation. Cyanopeptolin 1020 inhibits trypsin with an IC_50_ of 670 pM, with the arginine at position 2 playing a crucial role in binding [66]. The presence of phenylalanine at position 4 may enhance inhibitory potency. Following confirmation of trypsin inhibition, cyanopeptolin 1020 was tested in crustaceans, exhibiting an LC_50_ of 8.8 µM, comparable to MC toxicity. Cyanopeptolins also inhibit serine protease inhibitors, with an example being cyanopeptolin 954 displaying an IC_50_ of 45 nM [68]. Micropeptin T-20, isolated from a freshwater dam in Thailand, is currently the strongest known cyanopeptolin chymotrypsin inhibitor, with an IC_50_ of 2.5 nM [32]. Although their highest inhibitory activity is observed toward serine proteases, cyanopeptolins can also inhibit protein phosphatases and other peptidases.

Cyanopeptolin SS has a disulphated glyceric acid at positions 7 and 8, which was found to have an IC_50_ value below 10 µM for both PP1A and PP2A [45]. Micropeptin SF909 was extracted from a *Microsystis* bloom in Israel and tested on aminopeptidase N and cytosolic peptidase, where the IC_50_ values were 4.2 and 4.7 µM, respectively [35]. Furthermore, ecotoxicological studies indicate that cyanopeptolins 1007, 1020, and 1041 are toxic to nematodes, though their overall toxicity is lower than that of anabaenopeptins [69]. Cyanopeptolin 1020 was also identified as a neurotoxin to zebrafish embryos as it induced DNA damage [70]. Torres et al. tested cyanobacterial strains lacking MCs on zebrafish larvae and found cardiovascular abnormalities and, in some cases, lethal toxicity. Micropeptin K139 was hypothesized as a key toxic component [71].

Freshwater cyanopeptolins are produced by various cyanobacterial genera, including *Anabaenopsis*, *Microcystis*, *Woronichinia*, *Dolichospermum*, and *Planktothrix*. Similar to anabaenopeptins, their biosynthesis is directed by nonribosomal peptide synthetase (NRPS) gene clusters, specifically the *mcn* gene [60]. Cyanopeptolins exhibit extensive structural diversity, particularly within *Planktothrix* strains. In a Canadian lake, 80 unique cyanopeptolins were identified, from three different *Planktothrix* strains, producing a diverse array of variants simultaneously [62]. A drinking water reservoir supplying 45 mL/d experienced a cyanobacterial bloom dominated by *Anabaenopsis*, where anabaenopeptilide 202A was detected as the most abundant cyanopeptide, reaching concentrations of 22 ppb [42]. Beversdorf et al. monitored cyanopeptolins in raw water entering a drinking water treatment plant and detected cyanopeptolin 1007 at concentrations of up to 14.4 ppb over the course of their study [44]. Additionally, cyanopeptolin 1020, a potent trypsin inhibitor, was found at 7.1 ppb in source water, which is well above its toxicological threshold for enzyme inhibition [39].

These findings highlight the widespread presence of cyanopeptolins in freshwater systems and potential toxicity. Their toxicity has mainly been attributed to positions 2 and 3, which contain Ahp. Cyanopeptolins will continue to be found in freshwater sources and highlight the need for further research into their fate after cell lysis.

### 2.3. Aeruginosamide’s Structure, Toxicity, and Prevalence

**Figure 4:**
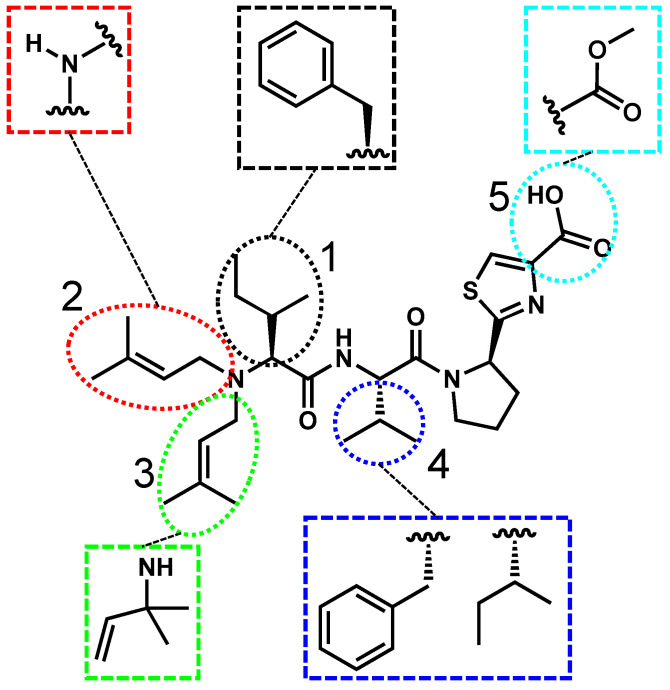
Aeruginosamide A is used as a representative structure for all freshwater aeruginosamides. The variable residues are shown in the colored boxes, and the positions are numbered 1–5.

Aeruginosamides are linear tetrapeptides or pentapeptides, notable for their prenylated N-terminus, pyrrolidine, and thiazole moieties (Figure 4). Although relatively few freshwater aeruginosamides have been fully characterized, existing data suggest variability at five positions. Proteinogenic residues such as isoleucine and phenylalanine have been identified at positions 1 and 4 [28]. Structural differences between congeners include an additional prenyl group at position 2 in aeruginosamide A, whereas aeruginosamide B features only hydrogen at this site [72]. Additionally, aeruginosamide B exhibits a methylated C-terminus at position 4, where the current literature has shown aeruginosamide A in a methylated form and a nonmethylated form [28,72]. Aeruginosamide C differs from aeruginosamide B as it has another peptide bond between the pyrrolidine and thiazole moieties [73].

Research on the toxicology of aeruginosamides remains limited, though available studies suggest cytotoxic activity against cancer cell lines. Lawton et al. were the first to report the isolation of aeruginosamide, subsequently demonstrating its cytotoxicity in A2790 human ovarian tumor cells (ID_50_ = 2.9 µM) and K562 human leukemia cells (ID_50_ = 5.2 µM) [28]. However, no other studies have demonstrated the effects of freshwater aeruginosamides on enzyme inhibition, general cytotoxicity, or toxicity in aquatic organisms. A recent study on aeruginosamides from brackish water indicated cytotoxicity against human breast cancer cells and potential inhibition of cytochrome P450 enzymes [72]. Despite these reports, the specific mode of action of aeruginosamides remains unknown, and their broader toxicological impacts are largely uncharacterized.

Aeruginosamides are synthesized through the cyanobactin biosynthetic pathway, a ribosomally synthesized and post-translationally modified peptide (RiPP) system. Within *Microcystis*, aeruginosamide production is specifically linked to the *age* gene within the cyanobactin cluster [74]. Freshwater aeruginosamides have primarily been identified in the genera *Microcystis*, *Oscillatoria*, and *Planktothrix* [73]. Despite their presence in multiple cyanobacterial genera, little is known about the aeruginosamide prevalence in freshwater systems. In one study, aeruginosamides B and C were detected at intracellular concentrations of 9.4 ppb and 0.863 ppb, respectively [36].

Aeruginosamides are one of the least studied cyanopeptides, so there is little information regarding their toxicity and prevalence. There is a need for toxicology studies and comprehensive field studies quantifying aeruginosamide extracellular concentrations.

### 2.4. Aeruginosin’s Structure, Toxicity, and Prevalence

**Figure 5:**
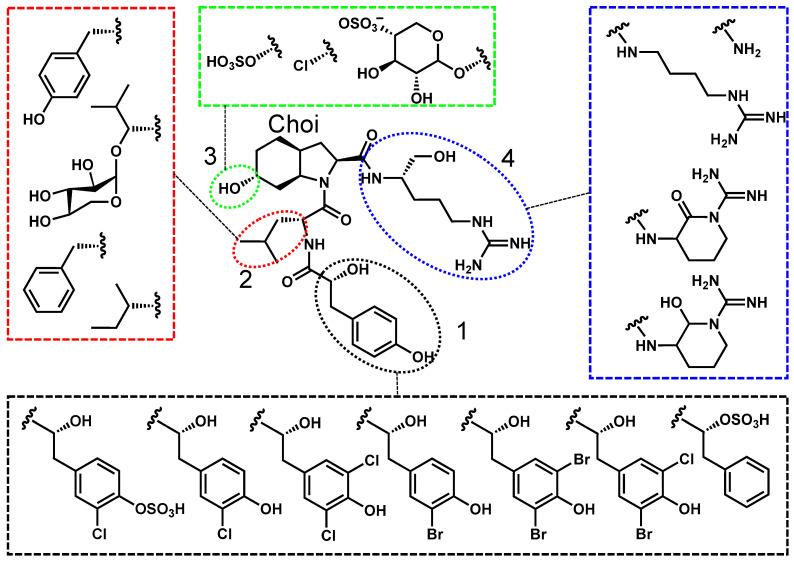
Aeruginosin 298A is used as a representative structure for all freshwater aeruginosins. The variable residues are shown in the colored boxes, and the positions are numbered 1–4.

Aeruginosins, also known as microcins and oscillarins, are linear tetrapeptides, structurally defined by four variable positions (Figure 5). The first position of interest is position 3, which contains 2-carboxy-6-hydroxyoctahydroindole (Choi), a non-proteinogenic amino acid that is essential for toxicity. The Choi moiety itself is frequently modified to a sulfated derivative, further increasing structural diversity [47]. In position 1, the N-terminus frequently features hydroxyl-phenyl lactic acid (Hpla) and halogenated variation, as seen in aeruginosin 298-A [29,75,76]. Position 2 varies between proteogenic (e.g., tyrosine, phenylalanine, isoleucine) and non-proteinogenic residues (e.g., hydroxyleucine xylopyranose) [77]. At position 4, the C-terminus contains an arginine derivative, which is essential for protease inhibition.

Aeruginosins are well known for their potent inhibition of serine proteases. Aeruginosin 98A, which features a chlorinated and sulfated position 1 and an argininal group at position 4, strongly inhibits trypsin, thrombin, and plasmin, with IC_50_ values of 0.4 nM, 0.03 nM, and 0.02 nM, respectively [47]. The presence of the argininal moiety at position 4 has been hypothesized to enhance its inhibitory potency [29]. Kohler et al. further investigated the structure–activity relationships of aeruginosins and found that the Choi group may play a critical role in protease binding. Specifically, aeruginosin 828A, which contains a sulfated Choi residue, exhibited low-nanomolar inhibition of trypsin and thrombin [78]. Subsequent ecotoxicity studies tested aeruginosin 828A on the freshwater crustacean *Thamnocephalus platyurus* (*T. platyurus*), where it exhibited an IC_50_ of 22.4 µM. The synthesis and repeated toxicity testing of aeruginosin 828A on *T. platyurus* was later confirmed by the Gademann group [79]. Additionally, Bownik et al. reported that *Chironomus aprilinus* larvae showed minimal adverse effects when exposed to aeruginosin B alone but exhibited increased oxygen consumption when exposed to aeruginosin B in combination with other cyanopeptides, suggesting that cyanopeptide mixtures may have additive or synergistic effects [21].

Aeruginosins are produced by cyanobacterial blooms containing *Microcystis*, *Nodularia*, and *Planktothrix*. Their biosynthesis is linked to a PKS/NRPS gene cluster, specifically the *aer* gene cluster [60]. Aeruginosin extracellular concentrations in freshwater systems remain largely uncharacterized. To date, reported aeruginosin concentrations have generally been in the sub-ppb range, though comprehensive prevalence studies are limited. Filatova et al. observed that aeruginosins accounted for 8% of the total cyanopeptides detected in their study, with aeruginosin 850 being the most abundant at 0.2 ppb [42]. Similarly, a Norwegian lake experiencing a *Planktothrix* bloom contained aeruginosin 583 at 0.2 ppb, making it the third most abundant cyanopeptide of that bloom, following MC-RR and anabaenopeptin B [46].

Aeruginosins are potent serine protease inhibitors and have been shown to affect aquatic life. The prevalence of these cyanopeptides currently seems low, but further studies are required.

### 2.5. Microginin’s Structure, Toxicity, and Prevalence

**Figure 6:**
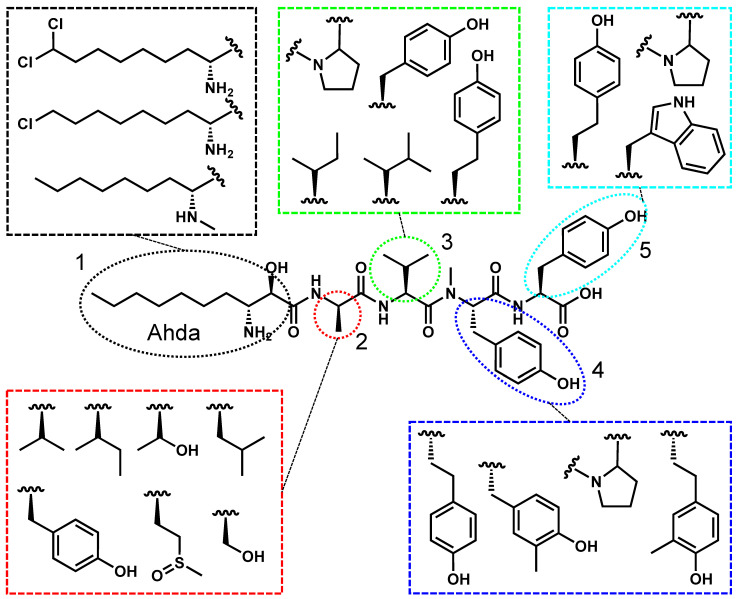
Microginin 713 is used as a representative structure for all freshwater microginins. The variable residues are shown in the colored boxes, and the positions are numbered 1–5.

Microginins, also known as oscillaginins, are linear peptides composed of four to six amino acids, exhibiting significant structural diversity (Figure 6). Unlike the previously stated cyanopeptides, each microginin position may be modified, though distinct structural patterns exist. Position 1 features a lipidated residue, 3-amino-(2-hydroxy)-decenoic acid (Ahda), which is critical for bioactivity and has been detected in singly and doubly chlorinated forms [80,81]. Positions 2 and 3 contain hydrophobic amino acids, at least one of which is N-methylated, while positions 4 and 5 often feature cyclic or aromatic residues, with tyrosine and homotyrosine being the most common [82,83,84].

Microginins are recognized for their peptidase and angiotensin-converting enzyme (ACE) inhibition. The first report of microginin shows inhibition of ACE with an IC_50_ value of 9.8 µM [51]. Lifshits et al. reported that microginin GH787, which is monochlorinated at position 1, exhibited an IC_50_ of 7.7 µM against aminopeptidase N [48]. Similarly, Ferreira et al. found that microginins displayed comparable inhibition capabilities to the known aminopeptidase N inhibitor, amastatin. Their findings suggest that the amine at position 1 plays a crucial role in binding at the Zn^2+^ interaction site, reinforcing the importance of this structural feature for enzymatic inhibition [81]. From an ecotoxicity perspective, Bownik et al. demonstrated that exposure to microginins led to neurotransmission disruptions in *Brachionus calyciflorus*, though the observed toxicity was lower than that of anabaenopeptins and MCs [18]. Additionally, Bober et al. found that microginin-containing biomass elicited a biological response in *T. platyurus* and *Daphnia pulex*. However, subsequent testing confirmed that microginins did not inhibit serine proteases [85].

Microginins are produced by various cyanobacterial genera, including *Microcystis*, *Oscillatoria*, *Planktothrix*, and *Woronichinia* [51,86,87,88]. Their biosynthesis is directed by a polyketide synthase/nonribosomal peptide synthetase (PKS/NRPS) biosynthetic gene cluster known as *mic* [60]. Field studies have revealed that microginins can reach significant environmental concentrations. In a Greek lake producing multiple cyanopeptides, the most abundant metabolite detected was microginin T1, measured at 47 ppb. This variant features a chlorine modification at the terminus of the Ahda group [49]. A lower but still substantial concentration was detected in the USA, as microginin 690 was detected in 35.2% of the sampled lakes with a maximum concentration of 2.2 ppb [50]. A particularly striking finding comes from a drinking water reservoir in China that serves 40 million people, where cyanostatin B was detected at concentrations reaching 1.3 ppm, which is two orders of magnitude higher than any detected MC in the same body of water [39]. These high environmental concentrations and potential toxicity raise concerns for the potential persistence of microginins prior to and through DWTPs.

### 2.6. Cyclamide’s Structure, Toxicity, and Prevalence

**Figure 7:**
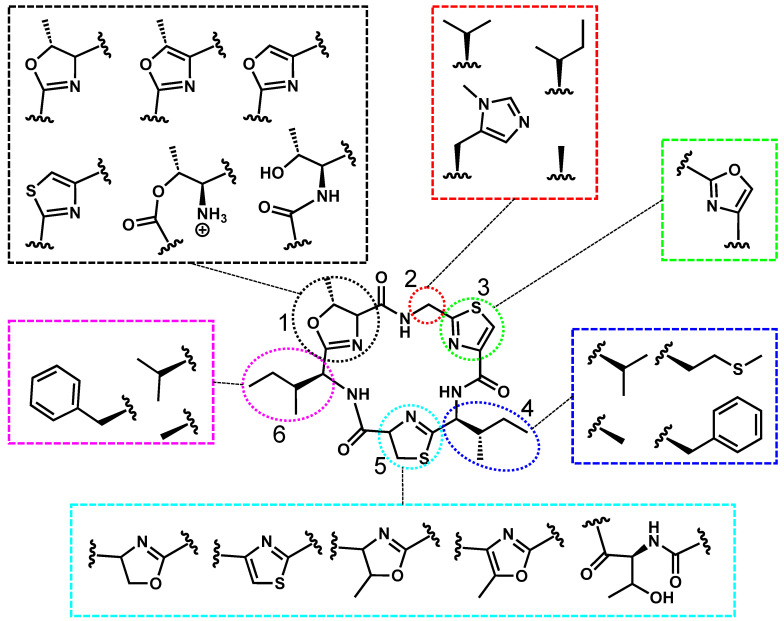
Aerucyclamide A is used as a representative structure for all freshwater cyclamides. The variable residues are shown in the colored boxes, and the positions are numbered 1–6.

Freshwater cyclamides are typically called aerucyclamides and microcyclamides, which are hexacyclic peptides, characterized by structural modifications at six distinct positions (Figure 7). Positions 1, 3, and 5 contain oxazole or thiazole derivatives, while positions 2, 4, and 6 typically feature nonpolar residues, such as isoleucine, valine, or phenylalanine. Structural differences among cyclamides include aerucyclamide A, which differs from aerucyclamide B by having two additional hydrogen atoms on the thiazole at position 5 [89]. Similarly, aerucyclamide D differs from aerucyclamide B by incorporating a phenylalanine at position 6 and a unique methionine residue at position 4 [90]. Although these structural modifications have been documented, the molecular features responsible for bioactivity remain unknown.

Cyclamides have demonstrated mild toxicity, particularly in relation to serine protease inhibition and cytotoxicity toward cancer cells. Microcyclamides exhibit an IC_50_ of 75 µM against chymotrypsin and have been shown to inhibit cancer cell proliferation by up to 36%. Conversely, inhibition of trypsin, thrombin, or elastase was not observed [52]. When first isolated, aerucyclamides A and B were tested for acute toxicity in the freshwater crustacean *T. platyurus*, where they exhibited LC_50_ values of 30.5 and 33.8 µM, respectively [89]. Further studies have explored their antiparasitic potential. Aerucyclamides A, C, and D demonstrated low micromolar IC_50_ values against *Plasmodium falciparum*, while aerucyclamide B showed even greater potency, with an IC_50_ of 700 nM. They also found microcyclamides exhibit toxicity in zebrafish embryos, where a 96 h exposure resulted in 62% lethality [37]. Against *Trypanosoma cruzi*, aerucyclamide C exhibited an IC_50_ of 9.2 µM [90].

Freshwater cyclamides belong to the cyanobactin class of cyanopeptides and are synthesized via the *mca* gene cluster [60]. They are predominantly associated with *Microcystis*. McDonald et al. found that aerucyclamide A-D represented the most abundant cyanopeptides in one *Microcystis* strain, whereas in another *Microcystis* strain, microcyclamide A was the dominant cyanopeptide [91]. MC-equivalent quantification methods have been used to estimate cyclamide abundance due to a lack of standards. For instance, the Janssen group reported that cyclamides accounted for 82% of the total cyanopeptides detected, with aerucyclamide A concentrations sixfold higher than those of MC-RR [92].

To date, freshwater cyclamides are understudied despite showing antiparasitic attributes and affecting aquatic life. Due to their estimated abundance, it is critical to know if cyclamides are deactivated before entering drinking water.

## 3. Degradation of Cyanopeptides

cHABs occur globally, often contaminating drinking water sources. Cyanopeptides are released into the water through cyanobacterial cell lysis or natural cell death, necessitating their removal or deactivation before or at DWTPs. Degradation of a compound is typically monitored by the absence of the original compound, while deactivation is when the compound no longer retains its ability to influence biological targets. In the context of cyanopeptide deactivation, it is typically attributed to chemical alteration of the residue(s) critical for binding. Prior to DWTPs, cyanopeptides are exposed to varying pHs, temperatures, light, and microorganisms that may degrade and potentially deactivate them entirely. If cyanopeptides prove to be environmentally stable and enter DWTPs, they will be exposed to conventional treatment strategies, including coagulation, sedimentation, and filtration, followed by disinfection, most commonly chlorination [93]. Chlorine is known to effectively deactivate MCs by modifying the Adda group, which is essential for their toxicity [94,95,96,97]. This section discusses the known degradation and potential deactivation pathways of the discussed cyanopeptides.

### 3.1. Degradation of Anabaenopeptins

Anabaenopeptin’s toxicity has been shown to be related to the ureido side chain at position 1 [43]. For deactivation to occur, this site must be chemically altered, or the general integrity of the interior cyclic ring must be compromised, resulting in loss of rigidity. Cyclic ring opening has been demonstrated to deactivate microcystin [94,95,96,97]. To investigate a pH and light degradation dependency, Natumi et al. exposed nine anabaenopeptins to pH ranges of 6.9–11.6 and UVA light [98]. A cHAB typically has a pH between 8 and 9 [50]. It was found that anabaenopeptins containing tyrosine residues exposed to UVA light at pH 9 or higher were susceptible to degradation. Anabaenopeptins B and C contain a tyrosine at position 5 and have half-lives of about 85 h, while anabaenopeptin A and oscillimide Y have half-lives of 48 and 24 h, respectively, which can be attributed to tyrosines or homotyrosines at position 5 and position 1 [98]. Anabaenopeptin A and oscillimide Y may be deactivated to a degree due to the alteration of the ureido linked tyrosine, but enzyme studies are needed to verify these compounds are deactivated.

Bober et al. further tested the degradation of anabaenopeptins regarding the pH and light but also included temperature. It was found that anabaenopeptin 899, which contains phenylalanine at position 1 and tyrosines at positions 5 and 6, degrades most efficiently when exposed to pH 3, 100 °C, and UV light [99]. Another deactivation pathway that is possible before DWTPs is degradation via microbes. Kato et al. determined that B-9, a bacterial strain that can degrade and linearize microcystin, does not degrade or linearize anabaenopeptin A [100]. However, Santos et al. determined that *Paucibacter toxinivorans* can linearize anabaenopeptins A and B along with microcystin [101].

Due to the relative stability under standard conditions, some anabaenopeptins may enter DWTPs, where they will be exposed to chlorination during the disinfectant stage. Little is known about how chlorination alters their structures, stability, and biological activity. Based on known chemical interactions, electron-rich residues such as cysteine, methionine, histidine, tryptophan, and tyrosine may be susceptible to chlorination, potentially disrupting their bioactivity. Conversely, cyanopeptides containing less reactive residues such as valine, leucine, isoleucine, and phenylalanine may remain largely unaffected by chlorination [102,103,104]. Based on chemical properties and the current known modes of action, anabaenopeptins containing electron-rich ureido side chains are most likely to be deactivated at DWTPs. However, the effects of these modifications on the biological activity of anabaenopeptins are not clear and warrant further investigation.

### 3.2. Degradation of Cyanopeptolins

Unlike anabaenopeptins, cyanopeptolins are potent serine protease inhibitors rather than carboxypeptidase inhibitors. Cyanopeptolin 1020, which exhibits picomolar inhibition of trypsin, relies on arginine at position 2 for binding [66]. It is hypothesized that deactivation of cyanopeptolin 1020 and other cyanopeptolins would result from a position 2 chemical alteration. When investigating cyanopeptolin 1081 (phenylalanine at position 2), it was found to be stable at room temperature while being exposed to visible light. However, it degraded by 32.3% over 3 h at pH 3. At pH 9, boiling the solution caused a 47% decrease [99]. Briand et al. investigated the degradation of cyanopeptides when exposed to bacteria commonly found in cHABs and observed that the signal for cyanopeptolin A (arginine at position 2) and 963A (tyrosine at position 2) could no longer be determined via mass spectrometry after 3 weeks [105]. For each of the previous degradation studies, the degradation products were not experimentally tested on enzymes, so full deactivation was not confirmed.

DWTP’s final stage is disinfecting the water and deactivating potential harmful sources from entering drinking water. Chlorination, a widely used disinfectant method, of MCs has been found to deactivate it, prohibiting it from inhibiting PP1 [106,107]. There have not been any studies to show the efficacy of chlorine in deactivating cyanopeptolins.

### 3.3. Degradation of Aeruginosamides

Early evidence shows aeruginosamides to be mildly cytotoxic against cancer cells, but their mode of action is not known. Additionally, there are currently no publications investigating how abiotic factors degrade and or deactivate aeruginosamides. These cyanopeptides differ structurally from anabaenopeptins and cyanopeptolins as they have linear structures. Their entire structure will be assumed as important for its cytotoxicity due to the lack of a known mode of action. In freshwater aeruginosamides, positions 1–4 have only been found to have nonpolar residues that are aliphatic or aromatic. These residues have been a sign of stability against temperature, pH, and light when compared to the previous cyanopeptides. Degradation and deactivation via bacteria and chlorination have not been investigated to the best of our knowledge. Based on the limited research in this area, we recommend studies into the degradation of aeruginosamides via temperature, pH, light, microorganisms, and disinfectants.

### 3.4. Degradation of Aeruginosins

The biological activity of aeruginosins is dictated by positions 3 and 4, where the Choi group (position 3) stabilizes binding while position 4 residues interact deeply within the binding pocket. Position 4 is commonly found in the form of argal, agmatine, or argininol [5]. Prior to DWTPs, aeruginosins are exposed to varying pHs, temperatures, light intensities, and bacteria. Natumi et al. attributed the tyrosine moiety on aeruginosin 298A for its ability to degrade at pH 9 with simulated sunlight [108]. The tyrosine chemical alteration at position 1 may not deactivate the cyanopeptide, as that residue is not critical for binding. There have been no explicit studies regarding temperature, microorganism degraders, or chlorination. The Choi group has been naturally found in chlorinated forms, suggesting that aeruginosins may undergo partial chlorination in the disinfection stage if it is not previously chlorinated. Positions 1 and 2, which frequently contain tyrosine, are also susceptible to chlorination, though some aeruginosins already exist in chlorinated forms (e.g., aeruginosin 828A with chloroleucine at position 2) and retain nanomolar protease inhibition [109]. The investigation of degradation pathways like temperature, pH, light, microorganisms, and disinfectants should be further explored.

### 3.5. Degradation of Microginins

Microginins function as peptidase inhibitors, with their inhibitory activity is linked to the amine attached to the Ahda group at position 1 [81]. Other positions (2–5) frequently contain electron-rich residues, such as tyrosine, methionine, and tryptophan [82,84]. Bober et al. tested variable abiotic conditions on microginins FR3, FR4, and 757. These microginins all had tyrosines at positions 4 and 5, while they differed slightly at positions 1 and 3. All three microginins were relatively stable when exposed to pH 3–9, temperatures 23–100 °C, and UV light. The biggest drop in concentration (~26%) for FR4 and 757 was seen while boiling for 1 h, while FR3 was unaffected [88,110]. There have not been any studies testing degrading organisms or disinfectants on microginins. Due to the observed stability of microginins to abiotic conditions, research into degraders and the fate of microginins through DWTPs is imperative.

### 3.6. Degradation of Cyclamides

The mode of action for cyclamides remains unclear, but they have been found to inhibit serine proteases and exhibit antiparasitic properties [37,90]. Structurally, positions 2, 4, and 6 primarily contain aliphatic residues, while positions 1, 3, and 5 contain oxazole and thiazole rings. Kato et al. observed a complete cyclic ring opening and loss of positions 4 and 5 of microcyclamide when exposing it to bacterial strain B-9 [100]. This loss of structure rigidity from ring opening has the potential to alter microcyclamides’ inhibition properties. Santos et al. investigated aerucyclamide A and D’s stability when exposed to non-*mlr* bacteria, *Paucibacter toxinivorans,* and found rapid degradation of the cyclamides [101]. Bacterial communities found from cHABs were also found to be a possible deactivation pathway, as aerucyclamides A, B, C, and D were degraded [105]. When considering the pH and light degradation, aerucyclamide A was found to be stable when exposed to pH 7–12 and UVA light for an hour [98]. On the contrary, aerucyclamide D, which contains a methionine at position 6, was highly reactive in sunlit lake water with a half-life of 3.4 h [108]. These electron-rich sites are potential sites for chlorination during disinfectant treatment. Investigations into the biological activity of degradation products should be carried out.

## 4. Conclusions

Cyanopeptides exhibit extensive structural diversity, influencing their biological activity, toxicity, and stability. Many of these cyanopeptides are detected at concentrations far exceeding MCs in freshwater sources, raising concerns about their potential persistence in the environment and DWTPs. Currently, no regulatory guidelines exist for the cyanopeptides mentioned in this review, despite their high prevalence in drinking water sources. This review highlights the known toxicities, prevalence, and degradation of six understudied cyanopeptides. The mentioned cyanopeptides experience a range of toxicity towards enzymes, cells, parasites, and aquatic life while also being found up to ppm levels that rival MCs. The current literature has shown that cyanopeptides with electron-rich residues are more susceptible to degradation, while electron-poor cyanopeptides may be environmentally stable. It is unclear if the current degradation pathways in the environment and DWTPs will deactivate the cyanopeptides before they enter drinking water. Due to the increased prevalence of cHABs and high environmental concentrations observed in the discussed cyanopeptides, future research into the degradation products’ toxicity is imperative to inform regulatory bodies and ensure public safety.

## Figures and Tables

**Table 1:** Each class of cyanopeptide with its known modes of action and respective IC_50_s. The table also shows the observed environmental concentrations found of each cyanopeptide in freshwater sources.

Cyanopeptide	Mode of Action	IC_50_ (µM)	Environmental Concentrations (ppb)	Ref.
Anabaenopeptin	Serine Proteases (Chymotrypsin, Elastase)	45, 14.3	1.5–1100	[40]
	Phosphatases (Protein Phosphatase 1A, 2A)	0.9, 1.3		[33,36,38,41,42]
	Carboxypeptidases (Carboxypeptidase A, B)	0.002, 0.0002		[34,43]
Cyanopeptolin	Serine Proteases (Trypsin, Chymotrypsin)	0.00067, 0.0025	7.1–22	[32]
	Phosphatases (Protein Phosphatase 1A, 2A)	>10, >10		[39,42,44,45]
	Peptidases (Aminopeptidase N, Cytosolic)	4.2, 4.7		[35]
Aeruginosamide	Cytotoxicity (human ovarian cancer, human K562 leukemia cells)	2.9, 5.2	0.893 *–9.4 *	[28,36]
Aeruginosin	Serine Protease (Trypsin, Thrombin, Plasmin)	0.4, 0.03, 0.02	0.2	[42,46,47]
Microginin	Peptidases (Aminopeptidase N, M)	7.7, 1.2	2.2–1324	[39,48,49,50]
	Angiotensin-Converting Enzyme	9.8		[51]
Cyclamide	Serine Proteases (Chymotrypsin)	75	N/A	[52]
	Antiparasitic (*Plasmodium falciparum* K1, *Trypanosoma cruzi*)	0.7, 1.1		[37]

* Intracellular concentration observed.

## Data Availability

No new data were created or analyzed in this study.

## Abbreviations

The following abbreviations are used in this manuscript:

cHAB	Cyanobacterial Harmful Algae Blooms
DWTP	Drinking Water Treatment Plant
MC	Microcystin
CPA	Carboxypeptidase A
CPB	Carboxypeptidase B
PP1	Protein Phosphatase 1
PP2A	Protein Phosphatase 2A
ACE	Angiotensin-Converting Enzyme
Ahp	3-amino-6-hydroxy-piperidone
Choi	2-carboxy-6-hydroxyoctahydroindole
Adda	(2S,3S,4E,6E,8S,9S)-3-amino-9-methoxy-2,6,8-trimethyl-10-phenyldeca-4,6-dienoic acid
Mdha	N-methyldehydroalanine
Adha	3-amino-(2-hydroxy)-decenoic acid
